# Assessing the functionality of an emergency obstetric referral system and continuum of care among public healthcare facilities in a low resource setting: an application of process mapping approach

**DOI:** 10.1186/s12913-021-06402-7

**Published:** 2021-04-29

**Authors:** Bernice Ofosu, Dan Ofori, Michael Ntumy, Kwaku Asah-Opoku, Theodore Boafor

**Affiliations:** 1 Department of Obstetrics & Gynaecology, KorleBu Teaching Hospital, Box 4236, Korle-Bu, Accra, Ghana; 2 University of Ghana Business School, Legon, Ghana; 3 University of Ghana Medical School, KorleBu, Accra, Ghana

**Keywords:** Emergency, Obstetric, Referral system, Women, Improve, Quality, Ghana, Access

## Abstract

**Background:**

Weak referral systems remain a major concern influencing timely access to the appropriate level of care during obstetric emergencies, particularly for Low-and Middle-Income Countries, including Ghana. It is a serious factor threatening the achievement of the maternal health Sustainable Development Goal. The objective of this study is to establish process details of emergency obstetric referral systems across different levels of public healthcare facilities to deepen understanding of systemic barriers and preliminary solutions in an urban district, using Ablekuma in Accra, Ghana as a case study.

**Methods:**

The study is an analytical cross-sectional study. Nine [1] targeted interviews were carried out for a three-week period in June and July 2019 after informed written consent with two [2] Obstetrics & Gynaecology consultants, two [2] Residents, one family physician, and four [3] Midwives managing emergency obstetric referral across different levels of facilities. Purposeful sampling technique was used to collect data that included a narration of the referral process, and challenges experienced with each step. Qualitative data was transcribed, coded by topics and thematically analysed. Transcribed narratives were used to draft a process map and analyze the defects within the emergency obstetric referral system.

**Results:**

Out of the 34 main activities in the referral process within the facilities, the study identified that 24 (70%) had a range of barriers in relation to communication, transport system, resources (space, equipment and physical structures), staffing (numbers and attitude), Healthcare providers (HCP) knowledge and compliance to referral policy and guideline, and financing for referral. These findings have implication on delay in accessing care. HCP suggested that strengthening communication and coordination, reviewing referral policy, training of all stakeholders and provision of essential resources would be beneficial.

**Conclusion:**

Our findings clearly establish that the emergency obstetric referral system between a typical teaching hospital in an urban district of Accra-Ghana and peripheral referral facilities, is functioning far below optimum levels. This suggests that the formulation and implementation of policies should be focused around structural and process improvement interventions, strengthening collaborations, communication and transport along the referral pathway. These suggestions are likely to ensure that women receive timely and quality care.

**Supplementary Information:**

The online version contains supplementary material available at 10.1186/s12913-021-06402-7.

## Background

Access to appropriate levels of care through referral systems is a complex process and remains a global public health concern. Appropriate and timely referral is crucial for women with emergency obstetric complications [4]. For a woman with emergency obstetric complication (s) especially patients with complications such as bleeding or severe hypertensive disorders, the World Health Organization (WHO), posits through its Safe Motherhood initiatives that the incapacity of the referral system to timely and safely transit to an appropriate level of care and expertise when complication occur may have devastating consequences on the maternal [2, 3, 5] and fetal outcomes including death. In general, a referral is characterized by multiple interrelated steps by an individual seeking specialist healthcare across different levels of care. The underlying assumption is that, in terms of diagnostics and therapeutics, higher-level facilities are better equipped than those from which the referral emanates.

It is estimated that in developing countries with limited resources, 15% of women who are pregnant, in labour, and in the postpartum period, are likely to develop an emergency complication [6]. Meanwhile, access to appropriate level of care remain a challenge particularly in Low-and Middle-Income countries (LMICs) [1, 4]. Considerable evidence suggests that adoption of frameworks, tools and techniques that deepen understanding of the structures and processes across the referral pathway better reveal defects which inform improvement efforts, likely to influence timely arrival to appropriate care [1, 7].

A number of authors [1, 7] have employed the use of different frameworks, tools, and techniques to unravel barriers of the referral system, but overall, these do not sufficiently assess the process details. An alternative approach is process mapping, a visualization technique which emerged from manufacturing and automobile industries but is increasingly being adopted in healthcare. One importance of process mapping is that, it breaks down complex processes into individual processes and illustrates where the steps in the process may diverge, occur in parallel, or exhibit gaps, uncertainties, specifying variations that may necessitate change or improvement strategies [8–10].

Although only a few studies have employed the use of process mapping [8, 11], including maternal health studies, their findings have revealed that the application of insights from process mapping helps to remarkably improve healthcare quality. Process mapping in general, is a potentially useful exercise but its use has been underutilized .. While rural systems are vulnerable, urban systems also bear significantly high burden of weak healthcare systems. To achieve Universal Health Coverage including Continuum of Care and the Sustainable Development Goal 3 on health, it is important to note this weakness in urban settings where a disproportionately high burden of maternal death exist [12, 13]; hence the need to identify systematic challenges in these settings. This leads to the central question: which areas can be targeted with interventions and improvement to make the referral system for women with emergency complication(s) across this context more efficient and productive, to ensure quality care?

In Ghana, one third of pregnant women (34%) reside beyond the clinically significant two-hour threshold from facilities likely to provide lifesaving obstetric care, influencing the timely arrival to appropriate care during obstetric emergencies [14]. The country adopted several initiatives to help improve access to appropriate level of care. This includes stratifying care across levels of care using a tiered model – Primary, Secondary and Tertiary; strengthening the capacity of facilities to care for emergency complications; and introducing a referral policy and guideline in 2012 [12]. These together with other interventions have led to a decline in one of the key maternal health outcome indicators, Maternal Mortality Ratio (MMR), from more than 500 estimated deaths per 100,000 live births in the year 2000 to 308 deaths per 100,000 live births in 2017. Despite this considerable reduction in MMR over the years, in Ghana, like many other sub-Saharan African countries that bear two thirds of the global maternal deaths, the latest estimate is still high, nearly 1.5 times higher than that of the global average and far from the Sustainable Development Goal (SDG) target of below 70 per 100,000 live births; to be achieved by the year 2030 [13]. Several studies [15, 16] in these contexts have shown that, timely arrival to appropriate care during obstetric complication is one major issue that hinders care outcome. This makes the need for a functional referral system critical and absolute. The objective of this study is to establish process details of emergency obstetric referral systems across different levels of public healthcare facilities to deepen understanding of systemic barriers and preliminary solutions in an urban district, using Ablekuma in Accra, Ghana as a case study.

## Methods

### Study design

This study employed a cross-sectional design approach. This study adopted the Interpretive/Constructive paradigm [17] to understand how the referral system operates as opposed to how it is intended. This enabled the researcher to explore the participants’ views on the referral systems and how they function instead of a theory-based approach. The researchers of this study recognised that frontline health workers and clinical leaders from varied backgrounds have their perspectives, beliefs, assumptions, and experiences that would contribute to the reality existing about the broader functionality of the referral system.

### Study setting

This study was conducted in three public health facilities in the Ablekuma district, specifically: in the Obstetrics and Gynaecology department of Korle Bu Teaching Hospital (KBTH) the third largest hospital in Africa and the largest tertiary hospital in Accra, Ghana; Mamprobi Polyclinic (MPC); and the Dansoman polyclinic (DPC). The setting was purposively selected to include public health facilities in an urban setting that provide primary, secondary and tertiary healthcare in the capital city of Ghana, Accra.

With regards to healthcare coverage, the Obstetric unit of KBTH provides specialized antenatal care, postnatal care to large and diverse population within and outside Accra and from lower levels of facilities, primary healthcare facilities, and district (secondary) hospitals both public and private. The total bed capacity for the hospital is over 2500 with 375 bed capacity for maternity services [18]. The hospital runs an antenatal clinic, has two labour wards and three theatres. The KBTH handles an estimated 27,128 new and old antenatal (ANC) attendance, 16,000 postnatal attendance and over 10,000 deliveries every year. Over 90% of cases are referred antepartum or intrapartum to KBTH and 70% of maternal deaths are referred cases [19].

**Fig. 1:**
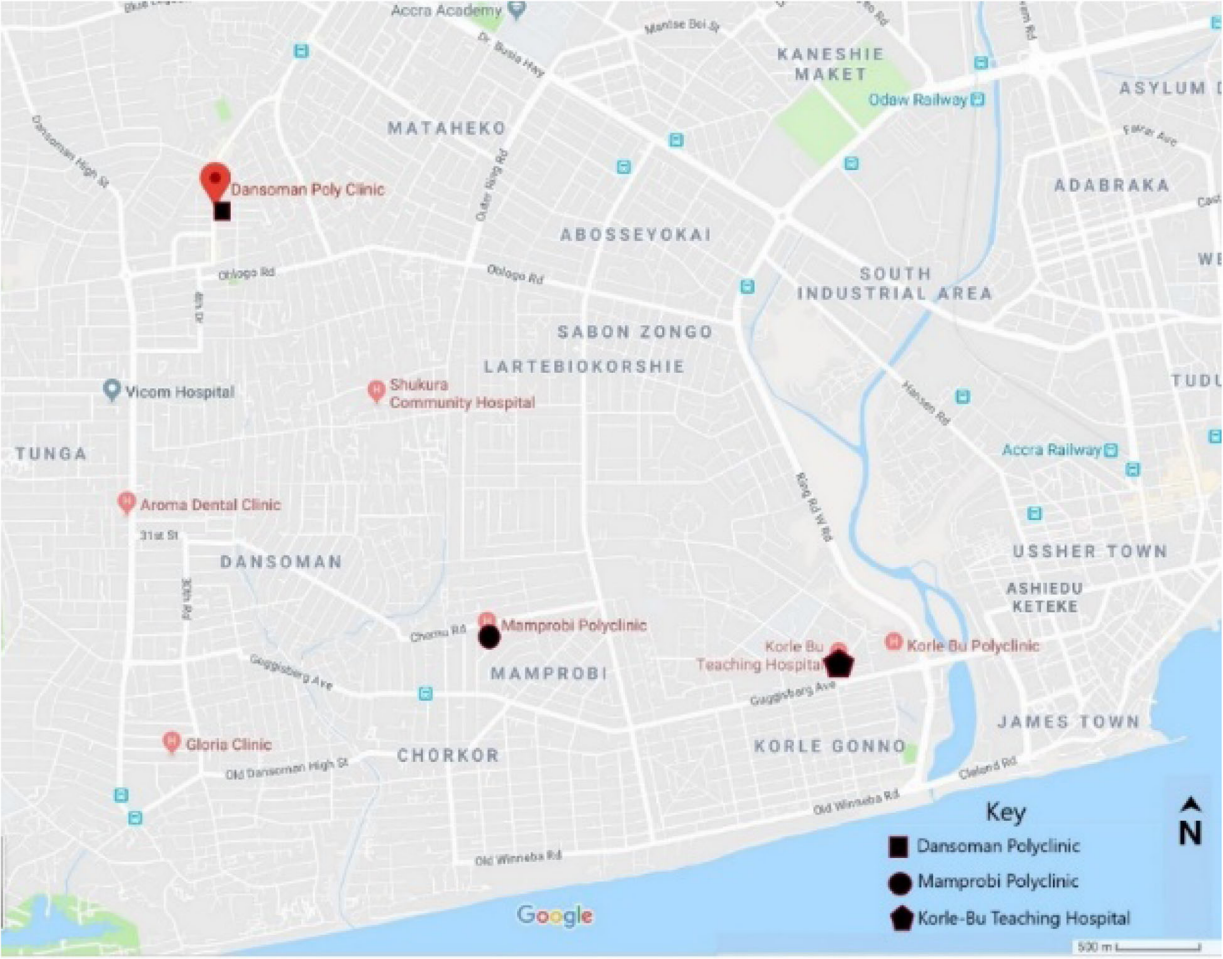
Map of Ablekuma district showing the facilities included in the study. Source: Google (n.d.)

For MPC and DPC, they reflect a cross-section of two levels of healthcare, primary and secondary. The KBTH and MPC are located in Ablekuma South whilst DPC is located in Ablekuma West. The map of the Ablekuma district is shown in Fig. 1**.** The DPC runs only antenatal clinics with one couch for ANC, 8 adult beds and 2 cots, a total capacity of 10 beds for the entire polyclinic. The Mamprobi Polyclinic runs an antenatal clinic, has a labour ward and an operating theatre. The annual ANC attendance in MPC is 18,677, the annual PNC attendance is 3089, annual deliveries is 2678 and total bed capacity is 45.

The Ablekuma South district has an estimated population of 213,914 [20]. The KBTH is about 2.66 km from MPC and about 6 Kilometers to DPC. The distance between DPC and MPC is about 4.65 km.

### Data collection

Healthcare providers executing varied professional roles were recruited using purposive sampling technique to obtain in-depth knowledge, individualized experience, and perception of the emergency obstetrics referral system. Data was collected using semi-structured key informant interview guide (see Referral Interview Guide) designed for this study to gather feedback about the process flow, existing barriers and recommendations for improvement. Data collection was undertaken by the primary researcher (BO) who has experience in undertaking qualitative public health and clinical research. Nine targeted interviews were conducted for a three-week period between June and July 2019 until saturation was met,ie, when the interviewer begun to hear the same responses over and over.

Participants were approached face-to-face by the data collector (BO) prior to the interview and were given written and verbal information about the study. After securing their written consent to participate in the study, a date, place and time was scheduled for the interview. All HCP approached accepted to participate in the study.

Two Obstetrician and Gynaecology consultants, two OBGY resident doctors, one Family Physician Specialist, and four Midwives across the three facilities in Ablekuma district in Accra, Ghana, were interviewed in English. Five were staff from KBTH, two from MPC and two from DPC. Interviewing involved the act of asking the respondents questions and audio-tape recording the responses and transcribing upon completion. Targeted interviews lasted on average 15 min. All interviews were conducted in person in a private office in the healthcare facility that would ensure privacy and convenience for the participants. The transcripts were randomly checked against the audio recordings for quality assurance purposes.

### Data analysis

A qualitative analysis was conducted in five phases. First, one researcher [BO] manually transcribed the interviews. This was reviewed by two other researchers [DO, MN] to eliminate bias, ensure consistency and check reliability. Second, we independently identified referral process related data in breadth and depth, from all nine interviews. Third, we used the transcribed narratives to draft a process map for the current emergency obstetric referral system within Ablekuma district, for each of the interviews. This was done initially for the lower-level facilities (MPC and DPC), then the higher-level facility (KBTH). Fourth, we refined and confirmed the process map ensuring that details from all interviews were reflected. Particular attention was paid to uncertainties about the referral process between the specified start and end points. That is, from when a woman with an obstetric complication comes to a lower-level facility to when discharged from a higher-level facility respectively. A process map was drawn for each interview and finally a summary process map drawn, incorporating all the individual process maps. The developed process map provided explicit visualisation of the referral process which was further analysed in two steps. First, we examined the uncertainties in the sequence of steps within the process and/or lack of systematised steps, gaps [that is, discrepancies of what the process is intended to be and what it actually is], bottlenecks within the process that cause delays before the next step occurs and inefficiencies [unnecessarily repeated steps leading to delays] to uncover potential areas for improvement broadly similar to approach used by . Key themes and sub themes were also discussed and reviewed by the five researches [BO, DO, MN, KAP, TB]. A meeting was later held with the participants to confirm the findings.

## Results

A total of nine [1] healthcare providers participated in the study: two Obstetrician and Gynaecology (OBGY) consultants, two OBGY resident doctors, one Family Physician Specialist, and four Midwives across the three facilities in Ablekuma district in Accra, Ghana. Five of the staff were from KBTH, two from MPC (a doctor and a midwife) and two from DPC (a doctor and a midwife). All interviews were carried out in English.

### Emerging themes

Overall, the main themes derived from the data analyses were (1) process flow/map (2) barriers (3) healthcare provider recommendations. The variation in the cadre of healthcare provider did not impact the response patterns.

### Process flow/map

**Fig. 2:**
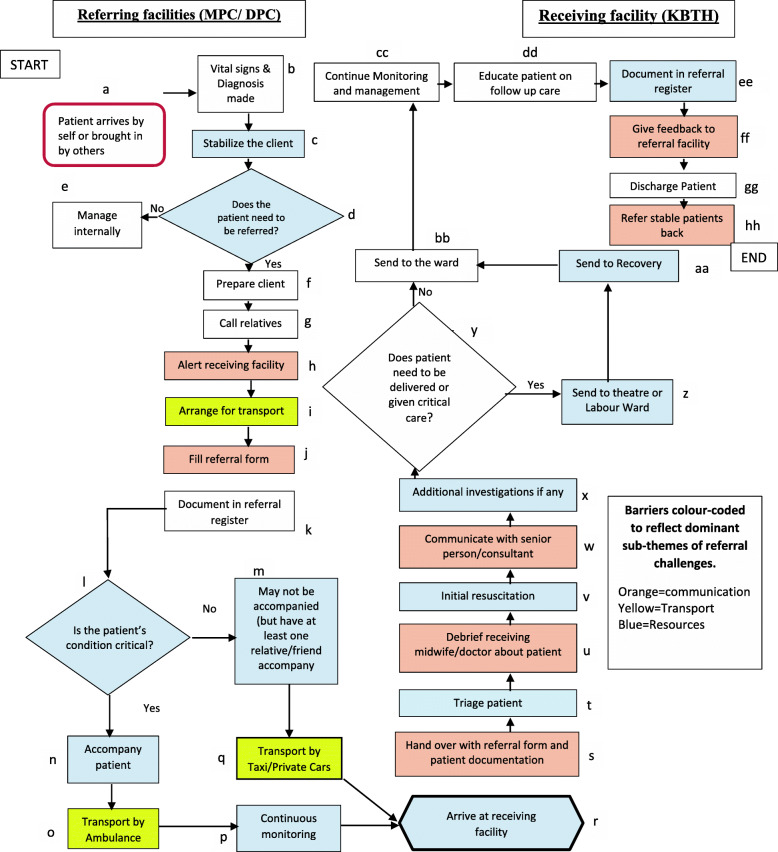
Process Map of Emergency Obstetric Referral System showing major barriers: (colored boxes) in referral processes in Ablekuma District (Attached)

Figure 2 above presents a process map designed on the basis of frontline healthcare workers’ narratives. It articulates the steps involved during a referral for women with emergency obstetric complications from lower-level facilities to a higher-level facility in Ablekuma district.

#### Lower-level facility

The referral process in this study starts when a lower-level facility receives a patient [a], they then make a diagnosis [b], stabilises the patient (c) and make a decision to refer [d] or not [e]. If a decision is made to refer, the referring facility prepares the patient and relatives which entails among others, counselling and securing intravenous access [f,g], calls or notifies the receiving institution [h], and the ambulance service providers. The ambulance service again calls the receiving institution to confirm their readiness to accept the case and also confirms with the referring facility the patient’s ability to pay for transport services [i]. When the higher level facility gives the green light, the lower level facility completes filling the referral form and document in the referral register [j]. A decision is made on whether a relative or a healthcare worker should accompany the patient [l]. The ambulance service driver then moves the ambulance from its station to the referring facility (MPC or DPC), then to the receiving facility accompanied either by patient relatives, a midwife, a nurse, a healthcare assistant or student nurse (s) [n] whilst the patients vital signs such as blood pressure, pulse and oxygen saturation are continuously monitored, depending on the case. [o, p]. In a situation where the ambulance service is unavailable, a taxi or a private car of relatives is used to carry the patient to a higher-level institution (KBTH) [q].

#### Higher-level facility

The higher-level period involves the following activities: The patient arrives in the facility [r], patient is handed over to the attending midwife at emergency unit of the receiving facility with the filled referral form and any other documentations [s], triaged [t], needed debriefing is done by the accompanying person(s) about patient’s previous management [u], patient is given initial resuscitation [v], a senior healthcare person is communicated to [w], additional investigations are requested if any [x] and patient is sent to the theatre or labour ward [z] or lying in-ward [bb] for further management. Patients who are sent to the theatre are sent to the recovery ward [aa] after which they are sent to the lying-in ward [bb], all patients on the ward [bb] have their vital signs continuously monitored and appropriate interventions given [cc], educated on follow up care [dd], and discharge documented [ee].. Feedback is then given to the referring facility [ff], patient discharged [gg], and stable patients referred back to the lower facility [hh].

### Barriers in the referral system

Out of the 34 major points/steps, findings suggest that 24 steps [c, d, h, i, j, l, m, n, o, p, q, r, s, t, u, v, w,, z, aa, bb, ee,ff, hh; noted using coloured coded shapes] often led to delays in moving from one step to the other while transferring the patient to an appropriate level of care, shown in Fig. 2. Barriers observed step-by-step in the referral process is detailed below:

### Stabilizing the client [c], deciding to refer [d], and manage internally [e]

In stabilizing the patient at the referring facility, healthcare workers at Dansoman Polyclinic (DPC) mentioned that there was no labour ward or a theatre at their facility. They had very limited space and lacked certain essential emergency medicines to provide care for women with emergency complication (s). Unlike DPC, Mamprobi Polyclinic (MPC) had a theatre, a labour ward and a space for antenatal clinic. However, health workers at MPC reported that there was no dedicated Obstetrician Gynaecologist (OBGY) readily available to undertake emergency surgeries should there be a need for one, at the time of collecting data. The MPC thus, relies on external OBGYs mainly from Korle Bu (the higher-level facility) to perform the surgeries in Mamprobi; that is if these OBGYs are available. Otherwise, MPC refers to a higher-level facility for further management. This situation often led to delays in deciding to refer the patient.

### Alerting receiving facility [h]

It was evident that staff from lower-level facilities feared their patients would be rejected when they call in advance to notify higher-level facilities. The fear and perceived bureaucracy deterred healthcare workers [e.g. midwives] at lower level facilities from notifying the receiving facility in advance before sending patients. One doctor from the receiving facility noted:

*“ … , some of these facilities just send patients without informing the recipient facility because sometimes the recipient facility do not have space, do not have bed and they think that if they are told there is no bed, they would have to contend with the difficulties that they are not prepared for, so they would rather want to stampede and just come without informing the recipient facility … ”* A2

Some healthcare workers from one of the lower facilities also noted:

*“ … sometimes you call and call [the receiving facility] and no one picks up the call … ” A9*

*“ … Most at times too, the call doesn’t go through and we are forced to move the client without informing a particular receiving end and you will be turned back because you haven’t called and this client may lose her life … sometimes if you don’t meet the one you spoke to on the phone the case may be rejected with the excuse that they were not informed. This usually happens during shift hours … ” A8*

### Arranging for transport [i]

The process involved in arranging for a means of transport for referrals for women with emergency obstetric complications seemed cumbersome. Additionally, there seemed to be a lack of trust among the referring institutions and stakeholders. For instance, the ambulance service providers always confirmed that the referring facility had called the higher facility even after they have been informed by the referring facility that they had already called. This led to bureaucracy and delay in dispatch of an ambulance to the facility that needs to refer the patient. As noted by a midwife:

*“ … The ambulance is also an issue because they always delay the process because they also confirm whether the client will be able to pay for the service before they call you back to inform you that they have confirmed from the receiving end before you can prepare for the transfer … “*A7

Another midwife described a scenario:

*“ … So, you have called whichever hospital you want to refer to and after they have accepted then you call an ambulance. The ambulance will also ask if you have already called the receiving end after which they [ambulance service providers] will call the receiving end to confirm before they accept to come … ”* A8

Financing for ambulance services was also a challenge. Specifically, the cost involved when using an ambulance was relatively high. Most patients who needed an ambulance did not have the ability to pay. One midwife described the situation. She said:

*“ … sometimes getting an ambulance is a bit of challenge. You have to resort to the private ambulances and some of them do charge fees at higher rate … so if we assess the patient and she cannot afford, then we use the taxi.”* A9

### Filling of referral form [j]

It was observed that a standard referral form was used by the referring facilities. However, respondents from a receiving facility noted that most often, filling of the referral form is incomplete which affected continuity of care. It was therefore difficult to seek clarification on patient’s prior history or management. One doctor at the receiving facility narrated:

*“ … sometimes where the referral details are scanty, you may want to get in touch with the referring facility to get them to fill in the gaps. But it doesn’t happen because often we don’t have the numbers on the referral form … ”* A2

### Deciding if patient condition is critical [l] to accompanying patient [n]

It appeared that the assessment of patients’ condition before a decision is made to accompany patient or not was not optimum. Consequently, respondents from the receiving facility expressed their displeasure at seriously ill patients coming in unaccompanied or accompanied by a person who could not provide them with the needed information about the patient. This was confirmed by one of the referring facilities that, due to their numbers, they assess to see the seriousness to join the patient to the referring facility or not.

### Transporting patient [o, q] and continuous monitoring during transport [p]

The study gathered that unsuitable means of transport: taxi and private cars were frequently used to transport patients from a lower facility to a higher-level facility due to their availability. The healthcare workers reported that this affected adequate monitoring and proper handling of patients whilst in transit to a higher facility. Consequently, such patients arrived at the higher facility in a poor state of health and in some instances, some patients lose their lives before arrival. One midwife described the effect of the non-availability of the appropriate means of transport. She said:

*“ … let’s say you are coming with a taxi and a client needs oxygen. Automatically, there is no way you can give oxygen to a patient in a taxi especially if they refer without an ambulance … .”* A3

### Arriving at receiving unit (KBTH) [r] and triaging of patients [t]

Despite KBTH having a patient triaging system, almost all respondents in the receiving facility admitted that the structural design at their reception made it difficult for patients, their relatives and/or a new nurse to find the emergency area. They explained that relatives or accompanying midwives had to pick up a wheelchair and transport patient to the emergency. In some instances, patients who do not know what to do walked to the emergency or triage area; which was about 50 m from the entrance of the maternity unit. In a case where an ambulance is used, the patient is wheeled to the emergency. According to a respondent:

*“ … the other bit is lack of well written out guidelines pasted at the emergency and entrance of the facility. Because of this gap [no notices at the entrance] people by their own way either carry the patient to the emergency or if they have a wheelchair at the entrance, they basically put the patient in it to the emergency … ” A2*

Similarly, it was gathered that the workload at the receiving facility did not allow healthcare workers to sometimes attend to cases specified in referral notes as emergencies as recommended by the referral policy, leading to delay in providing care to the patient. As one midwife narrates:

*“ … a case I see as an emergency may not be seen as an emergency at the referral facility. The client may have to join the queue when she gets there and this may worsen their condition … ” - A9*

### Handing over with referral form and patient documentation [s], and debriefing receiving midwife/doctor about patient [u]

Handing over of patient from the referring facility to the receiving facility was a challenge in some instances due to incomplete filling of referral forms, incomplete patient documentation and patient being accompanied by persons who do not have sufficient information. However, it seemed prior notice to the higher facility facilitated smooth handing over. As narrated by one midwife:

*“ … If they are aware that this patient is coming, they will receive but if they are not, they will toss you around and eventually you will have to return with the patient … ” A6*

### Initial resuscitation [v]

It is assumed that KBTH as a tertiary facility should have adequate manpower, equipment and other resources. However, issues of shortage of equipment and supplies existed. In addition to this, staff attitude affected the higher-facility’s preparedness and responsiveness to providing initial resuscitation. For instance, a midwife at the receiving facility mentioned that:

*“ … the things [Equipment] are there but as to whether they are working by the time you will take up they don’t check. For instance, oxygen cylinder it is there, the flow meter and everything but they won’t check so a patient may come needing oxygen and they open and it is empty … ” A4*

### Communication with senior person/consultant [w]

It appeared that the infrastructure or gadgets supporting communication in these facilities were not efficient and reliable. In some instances, healthcare workers had to use their own mobile phones to make calls. One doctor mentioned that:

*“ … GoTa phones existed but they are not functioning now … , so the information flow is not as it should be for such patients … ” A1*

### Additional investigations if any [x]

The National Health Insurance scheme (NHIS) was reported to be inefficient by three interview respondents. This according to them delayed urgent diagnostic investigations.

### Sending patients to theatre [z], recovery [aa] and ward

At the receiving facility, two theatres were functioning. The third theatre was not functioning, due to the unavailability of patient monitors. As explained by a respondent at the receiving facility, an average of 11 caesarean sections and about two [2] emergency surgeries such as hysterectomies were done in a day. On average, it takes 90 min to perform a routine caesarean section and room turnover and double that to conduct and turnover a complicated obstetric surgery. Importantly, about 30 women were delivered in KBTH in a day. About 40 % of these women often required caesarean section. Meanwhile, there were only four beds in the recovery ward and in some instances one or more of these beds were occupied by a seriously ill patient for days.

### Document in referral register [ee]

At the receiving facility there was a book that captures all daily admissions to the hospital. This captured all cases whether referred or attendant at the KBTH. This suggested that no register was dedicated exclusively for referred cases. This made it difficult to easily retrieve information concerning referred cases.

### Give feedback to referral facility [ff]

Giving and receiving feedback allows for learning and continuous quality improvement. However, it seems to be non-existent as one doctor describes it:

*“ … feedback is virtually non- existent until now that a rapid response team even came up and that is done at random. It is with every case that is referred that there should be feedback. But that is not happening at all. So, the feedback is poor just as the referral itself … .” A1*

In the receiving facility, there seemed to be no dedicated people who had complete information about all referred patients. This made referring facility personnel who tried calling to follow-up frustrated. Unless informal calls were made to personal colleagues to get feedback in higher-level institutions or calling patients directly, the feedback was not obtained.

One midwife and a doctor from a referring facility respectively mentioned that:

*“ … the higher facilities do not call to inform us about the cases we refer to them. No, it doesn’t happen like that unless the patient has passed away then you will be called and informed of what had happened … it is the relatives or clients we usually call to get feedback from and not the facility … ” A8*

*“ … On our part yes, we seek feedback but let me say that it hasn’t been formalized … .” A7*

Some of the healthcare workers from a referring facility attributed the non-existence of feedback to indiscriminate referrals which led to workload. It also appeared that healthcare leadership’s efforts in ensuring that procedures and processes were followed were inadequate. This affected the attitude and motivation of staff in making efforts to give feedback as they perceived nothing will be done to improve the process. One doctor gave a reason for lack of feedback:

*“ … Part of it may be that when you spend time to do proper feedback it may not actually come to anything because there isn’t any channel of command on the ground to ensure that things are looked at the way it should be looked at and things are corrected the way it should be corrected. No audit of what is going on … What case do you refer? What is the response? There is nothing happening as I am aware of in the referral sources to push anyone to look at what you have written. There is even not a proper channel. the referral system is not working the way it should work. … the chain of command on the ground is not being adhered to. And people are not being held or sanctioned for not taking responsibility … ” A1*

### Referring stable patients back to lower facilities [hh]

Referring stable patients from higher-level facility back to lower-level facility seem to be non-existent. As one doctor noted:

*“ … when patients are referred and they come with certain conditions and they are stabilized, it should be possible for them to be referred back to their primary healthcare facilities … ... But that does not also happen so usually you have all of them eventually coming to Korle Bu … ”- A2*

### Other barriers

Other barriers which affected the process were also identified. These were mainly related to the referral policy and guidelines; and leadership and accountability.

i. *Non-availability of policies and procedure documents:* Referring institutions had displayed adopted referral guidelines and contact numbers of referral focal persons at vantage points. However, guidelines on obstetric referrals including emergencies were not visibly displayed in the receiving facility. This questioned if a well laid down process existed. These concerns were confirmed by statements made by two doctors in the receiving facility:
  *“ … Yes, there is a written book and policy on the referral processes, except that I will not be able to lay hands on that book now. It is there [referral policy] but unfortunately looking from then on it is not being followed because of the high turnover of residents and nurses so at the point they will not even remember there was something like that...” A1*
  *“...we don’t have a well laid out process a number of the things are by convention so most of the time, people are told that this is how the emergency functions when they come in, but we don’t have a well laid out thing which is probably written and pasted at vantage points for people to know that this is how the emergency is supposed to run … “A2*
ii. *Insufficient knowledge of policies by health workers:* It also appeared that healthcare workers did not have sufficient knowledge about the existence of referral policies. One midwife mentioned:
  *“ … Well, I don’t know if there is any protocol. All I know of is there is an exchange center where you call to find out if there is a space. Apart from that, I don’t know of any other … ” A3*
iii. *Leadership and Accountability:* “*… But the chain of command on the ground is not being adhered to … So, it [Referral System] is not working the way it should work …*” *A1*

Overall sub-themes that emerged during qualitative analysis as barriers encountered in the emergency obstetric referral system were in relation to: communication, transport and financing for referral system, resources (space, equipment and physical structures), staffing (numbers and attitude), referral policy and guideline.

Critical analysis of the process map revealed gaps, uncertain steps, inefficient steps, and bottlenecks. In terms of gaps, the referral process described by respondents broadly conforms to requirements in Ghana’s Ministry of Health Referral Policy Guideline document [12]. However, there were some discrepancies between what this guide is intended for and how the process is carried out. For instance, the two-way referral from higher facility to lower facility rarely happens [hh]. A directory of facilities and facility contacts were not available [h]. A completed referral form as indicated by respondents in the higher-level facility often does not accompany the patients [s]. Prior communication to persons at higher facilities is sometimes not done [h], leading to systems’ unpreparedness. Provision of feedback between facilities rarely exists [ff]. Continuous medical care during transport is not assured in most instances as patients often use taxis or other forms of transport [q, p]. A few uncertainties were also brought to bear through the developed process map. For instance, it was unclear who accompanies the patient to the next level of care [n]. Also, it was unclear what the next step was when the patient gets to the tertiary facility (KBTH) [q]. Similarly, some steps in the process were unnecessarily replicated. For instance, in situations where the midwife needs to refer a client, she calls the ambulance service to notify them about a patient needing transport. Likewise, she is required to call the higher-level facility to enquire about their readiness. Meanwhile, to confirm this, the ambulance driver also calls the higher-level facility to enquire about their readiness to receive the patient [h].

### Suggested recommendations

The HCP shared their views on what strategies could help improve the current emergency obstetric referral system. They suggested an audit of the current referral forms; organisation of referral focused meetings, assigning a trained and dedicated personnel to effectively coordinate and monitor communication (Table 1: Q1 to Q8); a review of the implementation of National Health Insurance Services (NHIS) to remove bottlenecks of healthcare financing for critical diagnostics during emergencies, ensuring financing mechanism for the ambulance services especially in emergencies (Table 1: Q10-Q13). In addition, HCW recommended review and institutionalization of referral policy, Standard Operating Procedures for all stakeholders including ambulance service drivers to reduce bureaucracy; periodic education on the referral policy (Table 1: Q14-Q16); strengthening accountability and leadership (Table 1: Q14-Q16). HCP also suggested human and infrastructural capacity building; making available essential medicines and equipment; and reviewing emergency obstetric care readiness of facilities to enable them carry out optimum Emergency Obstetric care when needed (Table 1: Q19-Q22).

**Table 1:** Socio-demographic and Institutional Characteristics of study respondents

Background Characteristics	Frequency (%)
**Position**	
Consultant	2 (22.2)
Resident	1 (11.1)
Family Physician	1 (11.1)
Midwife	5 (55.6)
**Respondents place of work**	
KBTH	5 (55.6)
MPC	2 (22.2)
DPC	2 (22.2)
TOTAL	9 (100.0)

KBTH*Korle Bu Teaching Hospital, DPC* Dansoman Polyclinic, MPC* Mamprobi Polyclinic

Source: Survey Data (2019)

These suggestions are illustrated in Table 2**.**

**Table 2:** Suggested recommendations for the improvement of the emergency obstetric referral system

Sub-theme	Quote	
Multidisciplinary and inter-intra facility Communication & Coordination	Q1	“there needs to be a coordination of the emergency care...the whole district or sub-region has to have a meeting to iron out systematically patient flow and communication...and see how best they can improve on the present …” A1 Doctor
	Q2	“… we can do the WhatsApp platform for the referral sources, that would be the beginning of some better approach...” A1 Doctor
	Q3	“… Encourage Korle Bu to join the Kybele (WhatsApp Platform) or Korle Bu should create a similar medium to facilitate the delivery of prompt service … A8 Midwife
	Q4	“… we need to audit current referral forms and get them in a state that would help with communication …” A2 Doctor
	Q5	Take advantage of technology like activating the Kybele (WhatsApp Platform) at the district level … A7 Doctor
	Q6	Make communication gadgets available to facilitate smooth communicate among centres before referral … A9 Midwife
	Q7	“… there needs to be a better coordination between the referral sources and experts within the Ghana health service as well as outside so cases are not just pushed unnecessarily as an emergency to clog up clinics and clog up the wards that are required by genuine emergencies …” A1 Doctor
	Q8	“… getting the referral forms in such a way that referral facility eventually after the care has been given can also write back to the referring centres … because patients are stable …” A2 Doctor
	Q9	“… certain consumables … and the health insurance system should be reviewed and well-coordinated to help patient get the best care at the shortest time … A1Doctor
Strengthening Transport System	Q10	“… If ambulance service would overlook where they have to confirm from the receiving end before attending to the emergency will help to minimize the delay process. The money issue too if it can be looked at again because not all client can afford to pay for their service …” A8 Midwife
	Q11	“… I think we should go back to the old days where every health facility has their own ambulance it will really help and also if Korle Bu cannot be on Kybele, the facility can have it own form of Kybele it makes it easier …” A7 Doctor
	Q12	“… Review the current Ambulance service charges … A8 Midwife
	Q13	“… ambulance availability should be ensured …” A9 Midwife
Policy/ Guidelines	Q14	“… I think we should really pay attention to our referral system by putting a strategy and getting people to understand it. We should look at it as nation and not by individual facilities …” A6 Doctor
	Q15	“… there has to be an outline of what cases needs to be referred and until it is flagged that yes you can send this case, too many cases are being dumbed …” A1 Doctor
	Q16	“… we need good notices, we should type out what the processes are supposed to be; what the standard operating procedures are for everybody patients, drivers, ambulance, and even the care givers so they can follow the procedures to improve the lives of the people …” A2 Doctor
Leadership /Accountability	Q17	“… so, if that is in place [sanctions for not taking responsibility], people would play by the policies that are on the ground. …” A1 Doctor
	Q18	“… Until we really have some transparency in the apportion of blame accordingly if there is substandard care. It is not going to be a system that would correct itself …” A1 Doctor
Resources (Human Equipment, Medicines and Infrastructure)	Q19	“… Redesigning the entrance … once a vehicle lands there should be someone that people can approach … and do resuscitation along the line …” A2 Doctor
	Q20	“… the facility [DPC] must get its own delivery ward, get an attached obstetrician if not permanent one we can easily call to assist when we have problem … and the emergency bed management should improve at the higher facilities …” A6 Doctor
	Q21	“… it will go a long way to help if at least if the major once gets their own obstetricians... The person will not be able to work 24/7 but will lessen the number of referrals …” A7 Doctor
	Q22	“… most drugs for pre-referral treatment be made readily available if not free and also make available logistics to work with …” A9 Midwife

## Discussion

In the midst of global efforts to achieve the SDG related to maternal health, identifying improvement opportunities has been of much interest [21, 22]. This study utilised process mapping developed from the perspective of frontline health workers to examine the functions of an emergency obstetric referral system in a Low-and Middle-Income country. It breaks down the complex steps of the overall referral system as a woman presenting with emergency obstetric complication transits from a lower level to a higher-level public healthcare facility, highlighting the barriers and preliminary solutions that can support future policy and improvement initiatives.

The results of our study reveal that the referral processes for both lower-level facilities were similar and the sequence of steps was generally logical. However, HCP thought that the current emergency obstetric referral system is not working as it should. For example, referral facilities hardly get feedbacks from the recipient facilities. Across the 34 major steps taken during emergency obstetric referral in the district, on average almost three-quarters of the steps had defects, and critical steps like stabilizing patients at the lower-level facility and initial resuscitation at the higher facility were affected. These revelations are a commonly reported phenomenon in LMICs [22–25]. Afari et al. in 2014 examined system-based bottlenecks related to emergency obstetric care in rural Ghana . A little over one third of the steps had challenges, relatively less than what was observed in this study, although studies consistently suggest that rural settings are more vulnerable and liable to experiencing more barriers to accessing healthcare [3, 26]. A possible explanation for the variation in the findings is the detail and components of the referral steps that were examined.

This study analysed more closely how each step of the referral process actually happens. In addition, discrepancies were observed in how the referral process actually functions and what the Ghana’s Ministry of Health Referral Policy Guideline [12] document proposes. This was mainly due to low level of knowledge about the policy’s content, poor attitude towards compliance, inadequate monitoring and supervision. Literature reveals that variations in a process affects productivity, utilisation of services and work flow [27]. As suggested by HCP in this study, unless leadership capacity is built to monitor and supervise process-oriented practices, staff trained on the policies and process, and accountability demanded, variations between policy and practice are less likely to be addressed. These are recommendations consistent with that of other authors [21, 28–33].

Poor communication among referral staff was strongly reported by all healthcare workers as a major challenge affecting the referral process during obstetric emergencies. These findings are consistent with two qualitative studies in Ghana [26], and a systematic review in India [33] where access, uptake and effective use of information technology such as electronic health records remain a challenge. These challenges impede effective communication between referral and recipient health facilities. Similar findings in other low resource settings [32, 34] also observed lack of coordination and feedback during referral. Non-adoption of a responsive and robust communication system in an increasingly complex health system may continue to have implications on quality of care [35], team cohesion and possibly create conflict due to miscommunication [36].

System of transport for emergency referral was also another major challenge as healthcare workers in higher institutions reported that patients arrived in taxis or private cars and in a few instances in ambulances unaccompanied or accompanied by staff and/or with relatives of the patient. These findings of poor, absent or inappropriate means of transport are similar to several studies conducted in Ghana [23, 34, 37] and other resource-limited countries [22, 25, 30, 32, 33, 38]. This issue may be due to the fact that no budgetary allocations were made to support people who could not afford services during the referrals process [22] or pregnant women did not have money to cover transportation fees to travel to a health facility [25, 39].

In our study, healthcare providers identified other factors requiring attention such as: unavailability of essential supplies; shortage and poor distribution of staff; poor staff attitude; lack of recognition and motivation for staff managing referrals; lack of space; structural issues at triage areas; workload; long waiting times; and financing for referral which may lead to delay in accessing quality care; which are findings similar to other studies [16, 22, 25, 32, 34, 39–42]. Several authors [7, 22, 25, 43] including the World Health Organisation [28] have emphasized that these components are critical for a well-functioning referral system. The healthcare practitioners in this study recommended among other things, strengthening multidisciplinary and inter-intra facility communication and coordination, strengthening transport systems, and equipping facilities with needed resources.

### Strengths and limitations of the study

This study focused entirely on the perspectives of frontline health care workers. Hence, we cannot exclude the possibility of healthcare workers under-reporting situation (s) they perceived to be undesirable. Another limitation of this study is its cross-sectional design, which limits observation of the referral system over time, hence results should be interpreted within this context. Finally, it is noteworthy that this study focused on a specific geographical location which limits the generalisability of the results. Despite the limitations, this study has identified challenges and preliminary solutions that are highly relevant for the target population, with a good chance that if addressed has a potential of making emergency obstetric referrals in Ablekuma, a typical urban district, more effective and efficient and with a cascading effect of significantly improving maternal and neonatal health in Ghana. Moreover, to our knowledge, this is the first study to use process mapping to assess this subject in a low resource urban setting. It corroborates previous findings highlighting the importance of process mapping with perspectives from different cadres of healthcare providers as researchers recognised it as a critical area that needs urgent improvement. The extent and depth of the problems suggest that unless drastic improvement efforts are channelled to tackle these issues, the objectives of the SDGs may not be realised.

## Conclusions

Understanding the processes across the referral pathway is key to revealing defects which will inform proactive measures that are likely to influence timely arrival to quality care. Process mapping using the perspective of frontline workers suggests that the emergency obstetric referral system in a typical urban district in Ghana, a resource limited country is functioning less than optimally required. This study highlights the need for policy makers and healthcare managers to understand the complexity of factors influencing the efficient and effective emergency obstetric referral system. Managers should therefore facilitate the formulation and implementation of policies focused around improving communication, transport systems; effective distribution, institutionalisation and use of referral policy & guidelines; and assuring financing for referrals especially in the wake of Universal Health Coverage. Future studies might analyze and explore the potential impact of process issues on maternal and fetal outcomes in low resource settings.

## Supplementary Information

**Additional file 1: Appendix A.** Interview Guide (Mamprobi And Dansoman Polyclinic). **Appendix B.** Interview Guide (Korle-Bu Teaching Hospital).

## Data Availability

The datasets analyzed in this study are available from the corresponding author on reasonable request. All methods were carried out in accordance with relevant guidelines and regulations.

## Abbreviations

EOC: Emergency Obstetric Care
MMR: Maternal Mortality Ratio
