# Supplementary material for: Assessing the functionality of an emergency obstetric referral system and continuum of care among public healthcare facilities in a low resource setting: an application of process mapping approach
Source: BMC Health Serv Res. 2021 Apr 29;21:402. doi: 10.1186/s12913-021-06402-7 (PMC8082760; doi:10.1186/s12913-021-06402-7)
Supplement: Supplementary file 1 — Additional file 1: Appendix A. Interview Guide (Mamprobi And Dansoman Polyclinic). Appendix B. Interview Guide (Korle-Bu Teaching Hospital). [file 12913_2021_6402_MOESM1_ESM.docx]

## **Appendix A: Interview Guide (Mamprobi And Dansoman Polyclinic)**

1. where do you usually refer patients to?
2. Describe the processes you go through when you decide to refer a woman with emergency obstetric complication from your facility to a higher institution like KBTH?

*Probe: Who decided that this process should be followed? Is this process you are sharing documented here in your facility?*

1. What challenges do you face with the current referral process when getting a woman with emergency obstetrics complications to a higher institution like KBTH?

*Probe: Any other challenge?*

1. Over the period you have been referring patients to KBTH, which part of the process do you think is the least successfully implemented? What contributed to this limited success (barriers)? *Probe:* *Which part of the process is easy and clear? Which part of the process do you think is not easy and clear?*
2. What would make it easier to referring obstetric emergencies to a higher institution like KBTH*?*
3. Do you have any recommendations on how the emergency obstetric referral system could be improved? IF SO, could you please tell me? *Probe: Do you have other suggestions?*
4. Is there anything else that you would like to tell me or recommend that we have not already covered?

Thank you for your time in contributing to this interview.

## **Appendix B: Interview Guide (Korle-Bu Teaching Hospital)**

1. Describe the processes you go through when you have to receive and provide care to a woman referred with emergency obstetric complication from other institutions?

*Probe: Who decided that this process should be followed? Is this process you are sharing documented here in your facility?*

1. What challenges do you face with the current referral process when woman with emergency obstetrics complications are referred to KBTH?

*Probe: Any other challenge?*

1. Over the period you have been receiving women with emergency obstetric complications from other institutions, which part of the processes within Korle-Bu do you think is the least successfully implemented? What contributed to this limited success (barriers)? *Probe: Which part of the process is easy and clear? Which part of the process do you think is not easy and clear?*
2. Do you have any recommendations on how the emergency obstetric referral system could be improved? If so, could you please tell me? *Probe: Do you have other suggestions?*
3. Is there anything else that you would like to tell me or recommend that we have not already covered?

Thank you for your time in contributing to this interview.
